# CHARMM Force Field Parameterization of Peroxisome Proliferator-Activated Receptor γ Ligands

**DOI:** 10.3390/ijms18010015

**Published:** 2016-12-22

**Authors:** Melina Mottin, Paulo C. T. Souza, Clarisse G. Ricci, Munir S. Skaf

**Affiliations:** 1 Institute of Chemistry, University of Campinas—UNICAMP, P.O. Box 6154, Campinas 13082-864, SP, Brazil; melinamottin@gmail.com (M.M.); paulocts@gmail.com (P.C.T.S.); cla.g.ricci@gmail.com (C.G.R.); 2 Faculty of Pharmacy, Federal University of Goias, Goiânia 74605-170, GO, Brazil; 3 Faculty of Mathematics and Natural Sciences, University of Groningen, Groningen 9747, AG, The Netherlands; 4 Department of Pharmacology, University of California San Diego—UCSD, San Diego, CA 92093, USA

**Keywords:** PPARγ ligands, nuclear receptor, CHARMM parameters, molecular dynamics, GQ16, SR1664

## Abstract

The peroxisome proliferator-activated receptor γ (PPARγ) ligands are important therapeutic drugs for the treatment of type 2 diabetes, obesity and cardiovascular diseases. In particular, partial agonists and non-agonists are interesting targets to reduce glucose levels, presenting few side effects in comparison to full agonists. In this work, we present a set of CHARMM-based parameters of a molecular mechanics force field for two PPARγ ligands, GQ16 and SR1664. GQ16 belongs to the thiazolidinedione class of drugs and it is a PPARγ partial agonist that has been shown to promote the “browning” of white adipose tissue. SR1664 is the precursor of the PPARγ non-agonist class of ligands that activates PPARγ in a non-classical manner. Here, we use quantum chemical calculations consistent with the CHARMM protocol to obtain bonded and non-bonded parameters, including partial atomic charges and effective torsion potentials for both molecules. The newly parameterized models were evaluated by examining the behavior of GQ16 and SR1664 free in water and bound to the ligand binding pocket of PPARγ using molecular dynamics simulations. The potential parameters derived here are readily transferable to a variety of pharmaceutical compounds and similar PPARγ ligands.

## 1. Introduction

The peroxisome proliferator-activated receptor γ (PPARγ) is an important nuclear receptor (NR) which plays a major role in the regulation of glucose and lipid metabolisms [1,2,3,4]. Therefore, it has implications in different metabolic disorders such as diabetes, obesity, and cardiovascular diseases [1,5]. PPARγ activity can be modulated by phosphorylation reactions [6,7,8,9] and by ligand-dependent activation [3,10,11,12,13].

PPARγ ligands can modulate the activity of this nuclear receptor via distinct mechanisms, promoting antidiabetic action. The classical ligand-dependent activation involves inducing conformational changes in the ligand binding domain (LBD), especially in the helix 12 (H12). This structural rearrangement leads to the dissociation of corepressors and the recruitment of coactivators. The coactivators can then recruit the transcriptional machinery to the target gene promoter [14]. Alternative mechanisms induced by ligands involve the stabilization of other key regions of the LBD receptor [11,12,15,16], such as the inhibition of the phosphorylation [6,8]. The serine 245 residue, also located in the PPARγ LBD, is a recently discovered phosphorylation site [8] and it can be modified by the enzymes Cyclin-Dependent Kinase 5 (Cdk5) [6,8] and Extracellular Signal-Regulated Kinase (ERK) [9].

The most well-known PPARγ ligand, rosiglitazone (RSG) [17], belongs to the first discovered class of drugs active to the isoform γ of PPAR, the thiazolidine-2,4-diones or the thiazolidinedione (TZD) class [18,19,20]. RSG is a full agonist that strongly activates PPARγ by the classic mechanism of activation (through direct contact with helix 12) [21]. RSG reduces systemic insulin resistance and coordinately activates adipogenesis, storing or mobilizing adipocytes as needed. Despite all the benefits, full agonists cause some deleterious side effects such as weight gain, edema and congestive heart failure due to increased adipogenesis and fluid retention [19]. Many of these drugs, originally approved by the U.S. Food and Drug Administration (FDA), were withdrawn from the market.

Much effort has been invested in reducing the adverse effects of PPARγ ligands, while retaining the insulin-sensitizing effects of the receptor. This has been achieved through partial agonists. An example of a partial agonist of the TZD class is GQ16 (5-(5-bromo-2-methoxy-benzylidene)-3-(4-methyl-benzyl)-thiazolidine-2,4-dione) [20,22]. In vitro experiments have shown that GQ16 leads to 50% of PPARγ activity compared to the full agonist RSG. Qualitative assays showed that GQ16 promotes blocking of S245 phosphorylation mediated by Cdk5, like RSG, but exhibits no deleterious effects such as weight gain or edema in animals [22]. Moreover, a recent study reported that GQ16 can increase brown adipose tissue (BAT) activity and induce the expression of thermogenesis-related genes in visceral white adipocyte tissue (WAT) [23]. These findings suggest that PPARγ activity might be modulated by partial agonists to induce WAT browning and treat obesity [23].

A recent discovery of a new class of ligands, called non-agonists, allowed decoupling the antidiabetic activity from the binding ability to increase the rates of transcription featuring agonism. The first ligand of this class, SR1664 ((*S*)-4′-((5-((1-(4-nitrophenyl)ethyl)carbamoyl)-1H-indol-1-yl)methyl)-(1,1′-biphenyl)-2-carboxylic acid), presents no agonist activity but produces antidiabetic effects in vitro, without fluid retention, weight gain and interference in bone formation [7,8]. SR1664 acts specifically on the inhibition of the phosphorylation of PPARγ by Cdk5 [6].

The mechanism of action of PPARγ ligands is still not fully understood, especially regarding the partial and non-agonist ligands. Different from other NRs, the PPARγ binding cavity is large, flexible and partially hydrated [12,24]. The comparison of different crystal structures also indicates the possibility of multiple binding modes for the same ligands, as observed from the structures of SR1664 bound to PPARγ [25,26]. Besides, the PPARγ pocket can accommodate flexible and even multiple small ligands (as fatty acids) [27,28]. These characteristics make the development of new drugs a challenge for computational approaches, such as virtual screening and docking assays. On the other hand, atomistic molecular dynamics (MD) computer simulations can successfully investigate the interaction between proteins and the ligands, as well as the hydration and dynamics of the binding pocket [29,30,31,32,33,34,35,36]. However, the MD simulations require adequate force field parameters for proper treatment of the molecular interactions. In the case of PPARγ ligands, the force field parameters of the full agonist RSG were previously obtained by our group [37].

**Figure 1:**
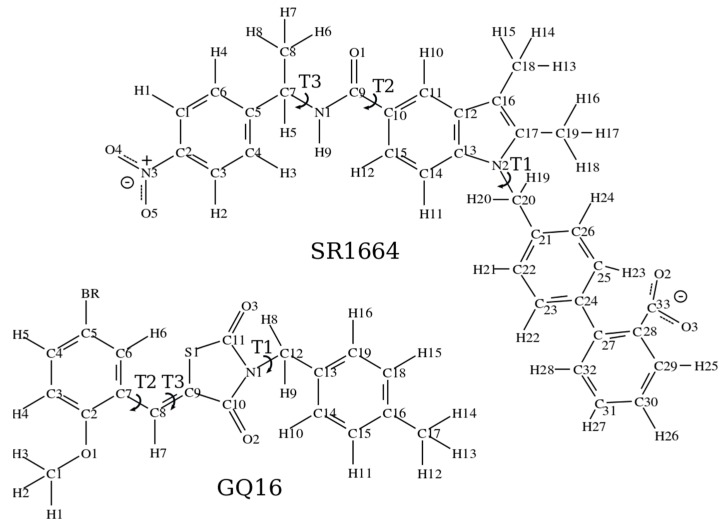
Chemical structures of the PPARγ ligands SR1664 and GQ16, indicating atom numbering and the parameterized torsions. The arc-shaped arrows indicate the torsion (T) bonds for which full revolution is possible.

In this work, we propose a set of CHARMM-based force field parameters for two PPARγ ligands, GQ16 and SR1664. Our goal is to generate the parameters of potential energy function for these molecules, which are suitable for further use in computer simulation studies. The CHARMM force field contains parameters available for bond stretching, angular distortions, Lennard-Jones energy and atom-atom distance for almost all atomic types. However, due to the lack of similar dihedrals available in CHARMM for the ligands of interest here, torsional potentials for some dihedral angles, depicted in Figure 1 as T1, T2 and T3, were parameterized to adequately describe the conformational properties of these molecules. Likewise, partial atomic charges were also parameterized.

## 2. Results and Discussion

The atomic charges calculated by Merz–Singh–Kollman for SR1664 and GQ16 are provided in Tables S1 and S2, respectively, according to the atom numbering of Figure 1. The charges are constrained to reproduce the molecular dipole moment. The charges were calculated from the fully optimized molecular geometries at the RHF/MP2 level in the quantum calculations. The total charge of SR1664 is −1, due to the charged carboxylate group, while for GQ16 the net charge is zero. For the purpose of computing the torsional potential energies, we have divided both ligands into smaller parts (according to the torsional angles to be calculated) and substituted larger side groups of the molecules with shorter surrogate fragments. This approach decreases the computational cost without compromising the essentials of the quantum chemical calculations regarding the torsional energies.

**Figure 2:**
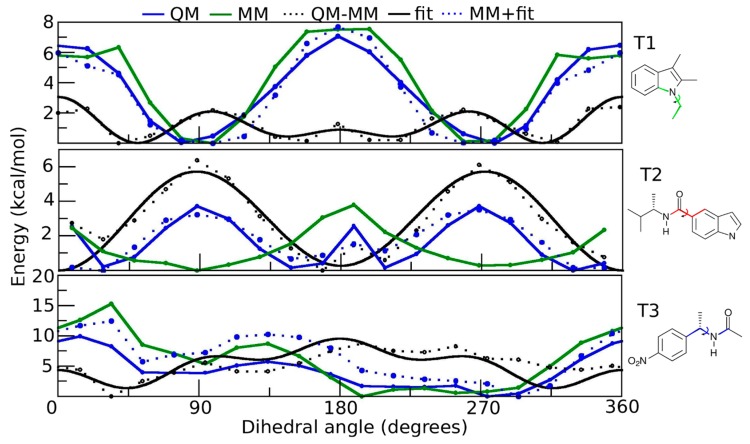
Potential energy curves generated for the torsions T1, T2, and T3 of the SR1664 molecule. The values of the potential energy computed by density functional theory are represented by the blue line (QM), whereas the corresponding values generated from the classical potential are represented by the green line (MM). The QM and MM energy differences are depicted by the black dotted line; the solid lines are the fitted dihedral potentials; the blue dotted lines are the sum MM and fitted dihedral potential. The structures on the right side of each graph depict the fragments used to calculate the potential energy curves (the calculated dihedrals are colored).

**Figure 3:**
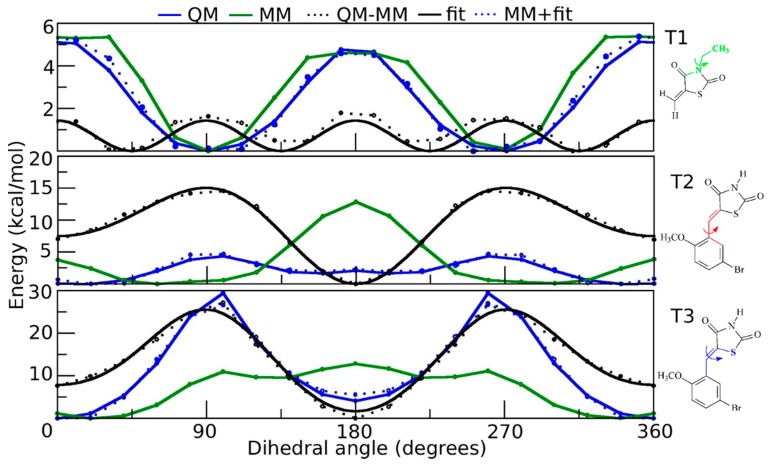
Potential energy curves generated for the torsions T1, T2, and T3 of the GQ16 molecule. The values of the potential energy computed by density functional theory are represented by the blue line (QM), whereas the corresponding values generated from the classical potential are represented by the green line (MM). The QM and MM energy differences are depicted by the black dotted line; the solid lines are the fitted dihedral potentials; the blue dotted lines are the sum MM and fitted dihedral potential. The structures on the right side of each graph depict the fragments used to calculate the potential energy curves (the calculated dihedrals are colored).

The potential energy surfaces (PES) obtained for the dihedral angles defined by T1, T2 and T3 shown in Figure 2 and Figure 3 describe the total dihedral angle potential for each bond rotation. However, the expression for the energy function considers all dihedral angles about a given bond. Therefore, the total constant *k*_φn_ was equally divided among all the involved dihedral angles. The best-fitting parameters for SR1664 and GQ16 are shown in Table 1 and Table 2, respectively, corresponding to the torsions T1, T2, and T3 obtained by adjusting the data depicted in Figure 2 and Figure 3.

The overall appearance of the quantum mechanics (QM) and molecular mechanics (MM) energy difference curves (black dotted lines) shown in Figure 2 and Figure 3 reflects factors such as the hybridization of the atoms involved and electronic resonance. Figure 2 shows the torsional PES for SR1664 and we can see from the QM-MM difference curve for T2 that the minima are centered at 0 and 180 degrees. This planar conformation is relative to the planar configuration of the peptide bond (the double bond feature between C-N atoms, the resonance between carbon from the carbonyl group and nitrogen). The QM-MM difference curve for T3 shows barriers at 0, 90, and 180 degrees relative to the steric hindrance between the ring and the nitrogen or oxygen atoms of the peptide bond. Figure 3 shows the PES for GQ16. The QM-MM difference curves for T2 and T3 exhibit minima at 0 and 180 degrees, corresponding to planar configurations. Nevertheless, the double bond is located in T3 (C8–C9), and not delocalized between C7–C8–C9, as indicated by comparing the barriers of the blue curves in T2 and T3.

**Figure 4:**
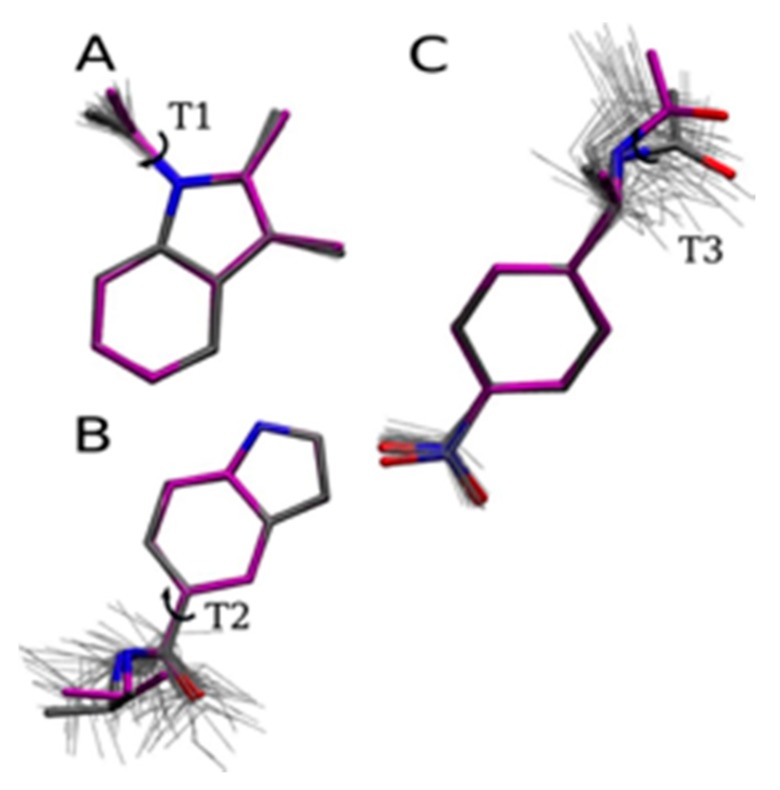
SR1664 fragment structures minimized at the MP2 level (carbon atoms in gray) and classically minimized (carbon atoms in purple), superposed with snapshots extracted from the MD simulations in vacuum (thin gray lines): (**A**) T1 fragment; (**B**) T2 fragment and (**C**) T3 fragment.

The optimized SR1664 fragments, used for the PES scan, are shown in Figure 4A–C, involving the T1, T2 and T3 dihedrals. There is a close similarity between the quantum and classical minimized structures. The quantum and classical structures are superposed on snapshots extracted from the MD simulations in a vacuum, showing an acceptable variation of parameterized dihedrals in MD simulations at 300 K.

**Figure 5:**
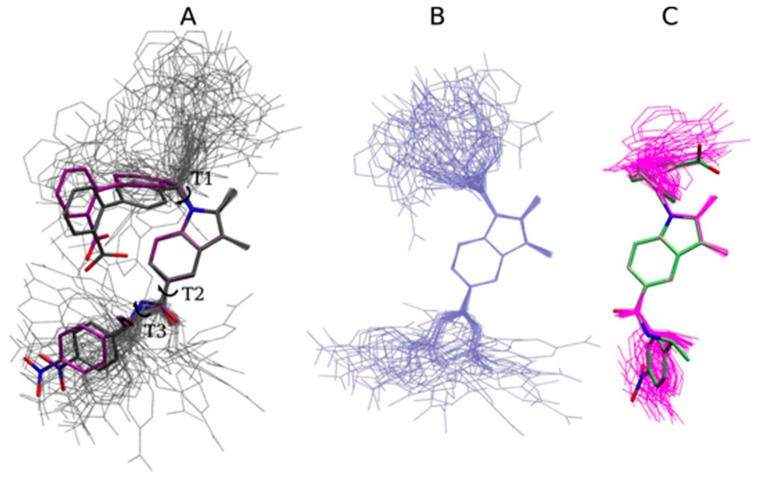
(**A**) SR1664 structures minimized at the MP2 level (carbon atoms in gray) and classically minimized (carbon atoms in purple), superposed with molecular snapshots of MD simulations in vacuum (thin gray lines); (**B**) molecular snapshots of MD simulations in water (thin light blue lines) and (**C**) molecular snapshots of MD simulations of PPARγ LBD (thin magenta lines) superposed with the crystallographic structure (carbon atoms in lime).

Figure 5 shows the entire SR1664 molecule, optimized by RHF/MP2 and MM, and the superposed molecular snapshots of SR1664 simulations in a vacuum at room temperature (Figure 5A) and in water (Figure 5B). In the quantum-optimized structure, an H-bond is formed between H9 and the carboxylate O2 atoms. The dihedral T1, which has a low torsional barrier, scans a wide range of configurations in vacuum as well as in water at room temperature. In the aqueous environment the intramolecular H-bond between H9 and O2 and other hydrophilic contacts are replaced by interactions with the solvent. Molecular dynamics simulations of SR1664 within the binding pocket of the protein (Figure 5C) reveal that non-bonded interactions between the protein and the ligand induce ligand conformational changes (RMSD plots for the protein backbone relative to the crystal structures are shown in Figure S1). This result shows the robustness of the developed parameters, allowing the ligand to behave differently depending on the environment. Because of the large volume of the PPARγ ligand binding pocket, it is likely that the ligand may assume other binding modes and conformations inside the LBD cavity. A detailed analysis of the ligand binding modes requires much longer MD runs for adequate sampling and deserves a separate study of its own.

**Figure 6:**
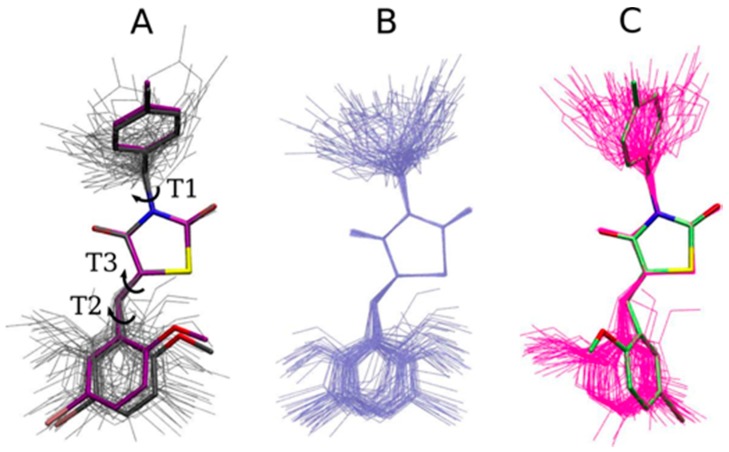
(**A**) Minimized GQ16 structures: QM (carbon atoms in gray) and MM (carbon atoms in purple) superposed with molecular snapshots from the GQ16 MD trajectory in vacuum (thin gray lines), aligning the central ring; (**B**) molecular snapshots of GQ16 MD simulations in water (thin light blue lines) and (**C**) molecular snapshots of GQ16 MD simulations in PPARγ LBD (thin magenta lines) superposed with crystallographic structure (carbon atoms in lime).

The optimized GQ16 structures and the molecular dynamics snapshots are shown in Figure 6. We can see that the quantum and classical optimizations are very similar (Figure 6A), indicating that the MM force field reproduces the quantum chemical ground state molecular conformation of GQ16 in vacuum. Figure 6A also shows the superposed molecular snapshots for GQ16 extracted from the MD trajectory in vacuum (aligning the central ring). The main changes are observed in the T2 and T1 dihedrals and this behavior is similar in the water environment (Figure 6B). The behavior of GQ16 in the LBD superposed on the crystallographic structure is shown in Figure 6C. We can see the protein’s influence, mainly in T2. The protein-ligand non-bonded interactions induce conformational changes in the ligand that make the ligand tend to reproduce the conformational features of the crystallographic structure, instead of fluctuating around the optimized structure. This result shows that the obtained GQ16 parameters are suitable to reproduce these differences in distinct chemical environments.

Ligand parameterization is a laborious process of several steps. Some servers/programs perform a fully automated fashion parameterization. However, after this automated process, further optimization of the parameters is often required. We tested the automated method for SR1664 and GQ16 molecules using the CHARMM General Force Field (CGenFF) program [38,39,40]. The CGenFF program performs atom typing and assignment of parameters and charges by analogy. A deterministic programmable decision tree assigns the atom type; the assignment of bonded parameters is based on substituting atom types in the definition of the desired parameter and the charges are assigned using an extended bond-charge increment scheme. The output file from CGenFF contains “penalties scores” associated with the partial charges and parameters. Penalties scores lower than 10 mean that the analogy is fair, penalties between 10 and 50 are usually associated with some basic validation, while scores higher than 50 indicate poor analogy and mandate extensive validation/optimization.

The automated fashion parameterization of both ligands, SR1664 and GQ16, generated parameters with high scores penalties, mainly related to the dihedrals scanned in this study. For SR1664, the higher parameter penalties obtained with CGenFF were 115.00, 71.9 and 72.9 for dihedrals C19–C17–N2–C20, C21–C20–N2–C17 and C21–C20–N2–C13, respectively. These dihedrals are related to the T1 torsion of SR1664 (see Figure 1). The higher charge penalty for SR1664 was less than 25. For GQ16, the higher parameters penalties were 172.00 for dihedral C10–C9–C8–C7 and S1–C9–C8–C7, related to the T3 torsion of GQ16 (see Figure 1), and the higher charge penalties were 58.138 for C10, 56.979 for C11 and 80.880 N1. Considering these, the parameters generated automatically have to be further optimized, especially for SR1664. For example, the CGenFF program considered completely different multiplicity and phases for the selected dihedral to C19–C17–N2–C20. This choice results in wrong conformations for the ligand, which can have severe consequences on the binding in the LBD pocket. These considerations show how important a proper force field parameterization could be, especially in the cases that the predictions by analogy clearly failed.

## 3. Materials and Methods

### 3.1. Parameterization of the PPARγ Ligands SR1664 and GQ16

The initial coordinates of GQ16 and SR1664 were obtained from the crystallographic structures with the LBD of PPARγ, available in the Protein Data Bank [41] under PDB IDs 3T03 [22] and 4R2U [25], respectively. The atoms are classified by analogy to atom types of the CHARMM22 all-atom force field for proteins [42]. The CHARMM force field functional form is expressed by Equation (1) as follows:

(1) $$\begin{array}{l} V=\sum_{\text{bonds}}k_{b}{\left(b-b_{0}\right)}^{2}+\sum_{\text{angles}}k_{\theta }{\left(\theta -\theta_{0}\right)}^{2}+\sum_{\text{Urey-Bradley}}k_{u}{\left(u-u_{0}\right)}^{2}\\ +\sum_{\text{diedrals}}k_{\phi n}\left[1+\text{cos}\left(n\phi -\delta_{n}\right)\right]+\sum_{\text{impropers}}k_{\omega }{\left(\omega -\omega_{0}\right)}^{2}\\ +\sum_{\text{non-bonded}}\left(-E_{\text{ij}}\left[{\left(\frac{R_{\text{min},\text{ij}}}{r_{\text{ij}}}\right)}^{12}-2{\left(\frac{R_{\text{min},\text{ij}}}{r_{\text{ij}}}\right)}^{6}\right]+\frac{q_{i}q_{j}}{4\pi \varepsilon_{0}r_{\text{ij}}}\right) \end{array}$$

where the force constants *k*_b_, *k*_θ_, *k*_u_, and *k*_ω_ were obtained by analogy with similar groups available in CHARMM and the respective values of the equilibrium bond lengths and angles obtained from the quantum chemical calculations. The nonbonded van der Waals interactions are described by 12–6 Lennard-Jones pair potentials, with parameters for each atomic type completely transferred from CHARMM. Non-bonded interactions were considered without any further scaling of the original non-bonded 1–4 terms of the CHARMM potential energy function (default).

For the torsional potentials of the dihedral angles, except T1, T2, T3 of SR1664 and T1, T2, T3 of GQ16 (Figure 1) we use parameters from CHARMM22 [42]. Some SR1664 parameters such as biphenyl ring, nitrobenzene and carboxylate were obtained from CHARMM General Force Field36, version 2b7 [38,39,40]. The bond lengths, bond angles and Lennard-Jones parameters of GQ16 bromine region were obtained from a molecule with similar bromine chemical environment [43]. Quantum chemical calculations were performed using Gaussian03 program [44]. Classical force field potential energies are computed using NAMD2.7 package, via NAMDEnergy plug-in of VMD [45]. All-atom protein set parameterization and molecular geometry optimizations were performed firstly at restricted Hartree-Fock (RHF), followed by Møller–Plesset second-order perturbation theory (MP2) with basis set 6-31G(d,p) for SR1664. We used the same procedure for GQ16, but due to bromine atom, the basis set used was 6-311+G(d,p). The MP2 level of theory is consistent with CHARMM parameterization [38] and has shown satisfactory agreement with experimental geometries for complex systems within an affordable computational effort. The partial atomic charges were obtained via a single point ab initio calculation at MP2 level (basis set: 6-31G(d,p) for the optimized structures of SR1664 and 6-311+G(d,p) for GQ16) and polarized continuum model (PCM), using Merz–Singh–Kollman scheme [46]. The same level of theory was used to determine selected dihedral energy profiles. The potential energy surfaces (PES) of the dihedral were obtained from full 360° rotational scans taken at 20° steps. At each 20° dihedral angle increment, the molecular geometry was fully relaxed, keeping fixed the selected dihedral angle. The molecular mechanics energy for each molecular conformation was computed using the NAMD 2.7 package. The corresponding values for the *k*_φn_, δ_n_ and *n* parameters were obtained by the nonlinear curve fitting. Consistently with the CHARMM force field, the phase angles were restricted to 0 or 180°.

### 3.2. Molecular Dynamics Simulations

Molecular dynamics simulations were performed to evaluate the developed parameters and the conformational behavior of the two ligands in vacuum, in water and in the LBD of PPARγ. All simulations were performed using NAMD2.7 [47] with CHARMM22/CMAP force field [42,48] for the protein, whereas water was described by TIP3P model [49]. The water and protein simulations were carried out in the NpT ensemble at 1 bar and 300 K, using the Langevin thermostat and the Langevin/Nosé-Hoover piston for the temperature and pressure control, respectively. Bonds involving apolar hydrogen atoms were constrained at their equilibrium values using the algorithm SHAKE [50] and a timestep of 2.0 fs was used for integrating the equations of motion. In vacuum, 1000 steps of ligand internal energy minimization were performed, followed by 4 ns MD simulations. For ligands in the aqueous environment, 4 ns simulations were carried out after equilibration with a single ligand solvated by a water shell of at least 15 Å thick. The simulations of ligand in water and in LBD of PPARγ were performed using periodic boundary conditions. Electrostatic interactions were computed using the Particle Mesh Ewald algorithm [51] and short-range interactions were truncated at a cutoff radius of 12 Å. The PPARγ-ligand systems were firstly prepared in a sequence of minimization steps and MD runs, keeping some constraints followed by production MD simulations without any constraints.

For PPARγ-SR1664 and PPARγ-GQ16 systems, we performed minimization steps followed by MD keeping the ligand and the protein fixed; minimization steps followed by MD keeping only the protein alpha carbons fixed. After these preparation steps, MD trajectories of 5.0 ns were generated without any constraints.

## 4. Conclusions

We developed a robust CHARMM-based model for two nuclear receptor ligands, SR1664, a PPARγ non-agonist, and GQ16, a PPARγ partial agonist. These ligands have relevant pharmaceutical applications in the treatment of type 2 diabetes and are developed focusing on reducing the side effects caused by full agonists. The proposed force field enables MD studies of the interactions of these molecules with proteins or biomolecular systems, such as the nuclear receptor PPARγ, under CHARMM. We have focused on the energy profiles of the dihedral that ensure the characteristic flexibility of the molecules, and are significant factors for ligand association/dissociation mechanisms and other features related to ligand conformational adaptations. MD simulations are being carried out for the PPARγ-SR1664 and PPARγ-GQ16 complexes using these potentials, aiming to investigate the interactions between ligands and amino acids of the PPARγ LBD, which can help further our understanding of how these ligands activate the NR.

## Figures and Tables

**Table 1:** Parameters of the torsional rotations T1, T2, and T3 for the SR1664 molecule determined by fitting the potential energy curves shown in Figure 2.

Dihedral Angle		*k*_φn_ (kcal/mol)	*n*	δ (°)
T1	C13 N2 C20 C21	0.1795	1	0.0
		0.3623	3	0.0
		0.4022	4	0.0
T2	N1 C9 C10 C11	0.1000	1	180.0
		0.7100	2	180.0
T3	C5 C7 N1 C9	1.5000	1	180.0
		0.1900	2	0.0
		0.2000	3	0.0
		0.7000	4	0.0

**Table 2:** Parameters of the torsional rotations T1, T2, and T3 for the GQ16 molecule determined by fitting the potential energy curves shown in Figure 3.

Dihedral Angle		*k*_φn_ (kcal/mol)	*n*	δ (°)
T1	C11 N1 C12 C13	−0.3572	4	180.0
T2	C6 C7 C8 C9	2.8584	1	0.0
		5.6498	2	180.0
		0.8824	3	0.0
T3	S1 C9 C8 C7	2.4869	1	0.0
		10.3926	2	180.0
		0.6072	3	0.0
		0.8350	4	0.0

## Supplementary Materials

Supplementary materials can be found at www.mdpi.com/1422-0067/18/1/15/s1.
