# Supplementary material for: CHARMM Force Field Parameterization of Peroxisome Proliferator-Activated Receptor γ Ligands
Source: Int J Mol Sci. 2016 Dec 22;18(1):15. doi: 10.3390/ijms18010015 (PMC5297650; doi:10.3390/ijms18010015)
Supplement: Supplementary file 1 [file ijms-18-00015-s001.pdf]

Supplementary Material: CHARMM Force Field Parameterization of Peroxisome Proliferator−Activated Receptor γ Ligands

Melina Mottin, Paulo C. T. Souza, Clarisse G. Ricci and Munir S. Skaf

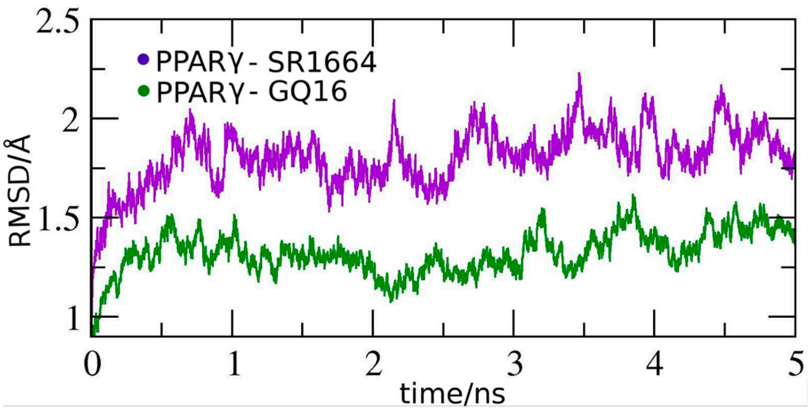

Figure S1. RMSD of PPARγ backbone for ligand−LBD complexes using the respective PPARγ crystallographic structures as a reference.

Table S1. Atom names, CHARMM atom types, and partial atomic charges derived in this work for the SR1664 molecule.

Atom	Atom Type	Charge (u.a.)	Atom	Atom Type	Charge (u.a.)
C1	CG2R61	−0.20	H14	HA	0.09
H1	HGR61	0.20	C15	CA	−0.04
N1	NTG	−0.47	H15	HA	0.09
O1	O	−0.74	C16	CY	−0.02
C2	CG2R61	0.09	H16	HA	0.13
H2	HGR61	0.20	C17	CA	0.12
N2	NY	−0.29	H17	HA	0.13
O2	OG2D2	−0.67	C18	CT3	−0.29
C3	CG2R61	−0.24	H18	HA	0.13
H3	HGR61	0.20	C19	CT3	−0.37
N3	NG2O1	0.71	H19	HA	0.06
O3	OG2D2	−0.79	C20	CT2	0.18
C4	CG2R61	−0.17	H20	HA	0.05
H4	HGR61	0.16	C21	CG2R61	−0.06
O4	OG2N1	−0.49	H21	HGR61	0.15
C5	CTG3	0.18	C22	CG2R61	−0.10
H5	HA	0.11	H22	HGR61	0.14
O5	OG2N1	−0.48	C23	CG2R61	−0.20
C6	CG2R61	−0.21	H23	HGR61	0.14
H6	HA	0.13	C24	CG2R67	0.07
C7	CT1	0.11	H24	HGR61	0.18
H7	HA	0.16	C25	CG2R61	−0.15
C8	CT3	−0.48	H25	HGR61	0.14
H8	HA	0.13	C26	CG2R61	−0.26
C9	C	0.77	H26	HGR61	0.16
H9	H	0.27	C27	CG2R67	0.17
C10	CA	−0.30	H27	HGR61	0.14
H10	HP	0.15	C28	CG2R61	−0.23
C11	CTG	−0.08	H28	HGR61	0.15
H11	HP	0.20	C29	CG2R61	−0.10
C12	CPT	−0.06	C30	CG2R61	−0.23
H12	HP	0.10	C31	CG2R61	−0.07
C13	CPT	0.11	C32	CG2R61	−0.29
H13	HA	0.10	C33	CG2O3	0.84
C14	CA	−0.25			

CTG, NTG, CTG3: new atomic types scanned; CG2O3, OG2D2, CG2R67: biphenyl negative carboxylate of CHARMM General Force Field 36; NG2O1, OG2N1: nitrobenzene CHARMM General Force Field 36.

Table S2. Atom names, CHARMM atom types, and partial atomic charges derived in this work for the GQ16 molecule.

Atom	Atomtype	Charge (u.a.)	Atom	Atomtype	Charge (u.a.)
C1	CT3	−0.24	C9	CTG2	−0.22
H1	HA	0.14	H9	HA	0.14
N1	NG	−0.26	C10	C	0.77
O1	OH1	−0.28	H10	HP	0.19
S1	S	−0.07	C11	C	0.61
C2	CA	0.30	H11	HP	0.21
H2	HA	0.14	C12	CT2	−0.16
O2	O	−0.63	H12	HA	0.12
C3	CA	−0.27	C13	CA	0.04
H3	HA	0.14	H13	HA	0.12
O3	O	−0.59	C14	CA	−0.15
C4	CA	−0.06	H14	HA	0.12
H4	HP	0.19	C15	CA	−0.40
C5	CA	−0.20	H15	HP	0.21
H5	HP	0.20	C16	CA	0.38
C6	CA1	−0.10	H16	HP	0.19
H6	HP	0.20	C17	CT3	−0.43
C7	CA	0.09	C18	CA	−0.40
H7	HAG	0.19	C19	CA	−0.15
C8	CTG1	−0.16	Br	BR	−0.08
H8	HA	0.14			

CTG1, CTG2, NG, HAG, CA1: new atomic types scanned.
